# Trifecta St. Jude medical® aortic valve in pulmonary position

**DOI:** 10.1080/20022727.2017.1299900

**Published:** 2017-05-01

**Authors:** Antonio F. Corno, Alan G. Dawson, Aidan P. Bolger, Branco Mimic, Suhair O. Shebani, Gregory J. Skinner, Simone Speggiorin

**Affiliations:** a Service of Paediatric and Congenital Cardiac Surgery, University Hospital Leicester, Glenfield Hospital, Leicester, UK; b Service of Adult Congenital Cardiology, University Hospital Leicester, Glenfield Hospital, Leicester, UK; c Service of Paediatric Cardiology, University Hospital Leicester, Glenfield Hospital, Leicester, UK

**Keywords:** Congenital heart defects, cardiac surgery, pulmonary valve, pulmonary valve regurgitation, right ventricle, surgical remodelling

## Abstract

**Introduction**: To evaluate an aortic pericardial valve for pulmonary valve (PV) regurgitation after repair of congenital heart defects.

**Methods**: From July 2012 to June 2016 71 patients, mean age 24 ± 13 years (four to years) underwent PV implantation of aortic pericardial valve, mean interval after previous repair = 21 ± 10 years (two to 47 years). Previous surgery at mean age 3.2 ± 7.2 years (one day to 49 years): tetralogy of Fallot repair in 83% (59/71), pulmonary valvotomy in 11% (8/71), relief of right ventricular outflow tract (RVOT) obstruction in 6% (4/71). Pre-operative echocardiography and MRI showed severe PV regurgitation in 97% (69/71), moderate in 3% (2/71) with associated RVOT obstruction. MRI and knowledge-based reconstruction 3D volumetry (KBR-3D-volumetry) showed mean PV regurgitation = 42 ± 9% (20–58%), mean indexed RV end-diastolic volume = 169 ± 33 (130–265) ml m^–2^ BSA and mean ejection fraction (EF) = 46 ± 8% (33–61%). Cardio-pulmonary exercise showed mean peak O_2_/uptake = 24 ± 8 ml kg^–1^ min^–1^ (14–45 ml kg^–1^ min^–1^), predicted max O_2_/uptake 66 ± 17% (26–97%). Pre-operative NYHA class was I in 17% (12/71) patients, II in 70% (50/71) and III in 13% (9/71).

**Results**: Mean cardio-pulmonary bypass duration was 95 ± 30ʹ (38–190ʹ), mean aortic cross-clamp in 23% (16/71) 46 ± 31ʹ (8–95ʹ), with 77% (55/71) implantations without aortic cross-clamp. Size of implanted PV: 21 mm in seven patients, 23 mm in 33, 25 mm in 23, and 27 mm in eight. The *z*-score of the implanted PV was −0.16 ± 0.80 (−1.6 to 2.5), effective orifice area indexed (for BSA) of native PV was 1.5 ± 0.2 (1.2 to –2.1) vs. implanted PV 1.2 ± 0.3 (0.76 to –2.5) (*p* = ns). In 76% (54/71) patients surgical RV modelling was associated. Mean duration of mechanical ventilation was 6 ± 5 h (0–26 h), mean ICU stay 21 ± 11 h (12–64 h), mean hospital stay 6 ± 3 days (three to 19 days). In mean follow-up = 25 ± 14 months (six to 53 months) there were no early/late deaths, no need for cardiac intervention/re-operation, no valve-related complications, thrombosis or endocarditis. Last echocardiography showed absent PV regurgitation in 87.3% (62/71) patients, trivial/mild degree in 11.3% (8/71), moderate degree in 1.45% (1/71), mean max peak velocity through RVOT 1.6 ± 0.4 (1.0–2.4) m s^–1^. Mean indexed RV end-diastolic volume at MRI/KBR-3D-volumetry was 96 ± 20 (63–151) ml m^–2^ BSA, lower than pre-operatively (*p* < 0.001), and mean EF = 55 ± 4% (49–61%), higher than pre-operatively (*p* < 0.05). Almost all patients (99% = 70/71) remain in NYHA class I, 1.45% = 1/71 in class II.

**Conclusion**: (a) Aortic pericardial valve is implantable in PV position with an easy and reproducible surgical technique; (b) valve size adequate for patient BSA can be implanted with simultaneous RV remodelling; (c) medium-term outcomes are good with maintained PV function, RV dimensions significantly reduced and EF significantly improved; (d) adequate valve size will allow later percutaneous valve-in-valve implantation.

## 1. Introduction

Surgical treatment for paediatric patients with congenital right ventricular outflow tract obstructions, including multi-level (sub-valvular, valvular and/or supra-valvular) pulmonary stenosis and tetralogy of Fallot, were already reported with excellent results a few decades ago.[1] Pulmonary valve (PV) regurgitation resulting from surgical repair, particularly after incision of the PV annulus and transannular patch for repair of tetralogy of Fallot or other right ventricular outflow tract obstruction with hypoplastic PV annulus, is generally quite well tolerated in the post-operative period, and it was initially thought to be a quite benign condition.[2] However, on longer-term follow-up and subsequent review, the presence of severe PV regurgitation was reported to be associated with exercise intolerance, heart failure, ventricular arrhythmias with syncopal episodes and sudden death.[3–11] These symptoms are related to right ventricular volume and/or pressure overload, inducing dilatation and dysfunction, and ultimately right ventricular failure;[5,9,10,12] sometimes the clinical conditions are further complicated by subsequent impairment of the left ventricular function.[9]

PV implantation, possibly associated with right ventricular surgical remodelling, has been shown to reduce the right ventricular end diastolic volume, thereby reducing the degree of right ventricular dilatation, and preventing abnormal right ventricular remodelling.[8,10,12–17] Patients with severe PV regurgitation can therefore benefit from this procedure to restore right ventricular volume and improve its function, preventing progression towards right ventricular failure.

While general agreement exists on the need for a PV implantation in the presence of severe PV regurgitation after surgical treatment for congenital heart defects, extensive debates accompany the following issues:

- timing of PV implantation, particularly in asymptomatic patients;[6–8,10,14–25]
- choice of the approach, between percutaneous interventional [6,26–33] versus surgical [8,10,12–19,34–38] PV implantation;
- choice of the most suitable valve;[3,34–48] and
- role of the surgical right ventricular remodelling.[49–51]

## 2. Aims

The purpose of this study is to assess the safety in terms of mortality and the early and mid-term outcomes of the use of the Trifecta St. Jude Medical® aortic valve used in the PV position for patients with PV regurgitation.

## 3. Methods

The hospital congenital cardiac surgical database was searched to identify all patients with previous surgery for congenital heart malformation involving the PV who underwent PV replacement with Trifecta St. Jude Medical® aortic valve in the four-year period from the first implant in July 2012 to June 2016.

The criteria for exclusion were: (a) patients with a mechanical PV replacement, alternative biological valve or biological valved conduit; and (b) patients with PV replacement as primary surgical procedure without previous surgery for congenital pulmonary valve or right ventricular outflow tract abnormality. Exclusion criteria were elicited by review of the patient’s hospital records.

### 3.1. Pulmonary valve implantation

PV implantation was performed in all patients through re-do median sternotomy. Cardiopulmonary bypass was established with aortic and right atrial cannulation, on a beating heart under normothermic conditions. Bicaval venous cannulation and aortic cross clamp were used in the presence of residual intra-cardiac defects requiring surgical closure. Femoral vessel cannulation was used at the beginning of the experience in anticipation of the presence of severe adhesions. If a transannular patch had been used, this was completely excised. The PV annulus was inspected and measured, and the largest Trifecta St. Jude Medical® valve, suitable for the z-score of the native PV of the patient, was selected and implanted with running sutures; the upper aspect of the prosthesis was attached to a heterologous pericardial patch enlarging the PV annulus. Right ventricular remodelling was performed when indicated, by resecting the non-contractile portion of the free right ventricular wall adjacent to the previously implanted transannular patch.

### 3.2. Data collection

Patient demographic and basic clinical information was recorded from the patients’ hospital records, including age, body weight, New York Heart Association (NYHA) classification, type and date of previous cardiac operations. Pre-operative investigations were reviewed, including echocardiogram with measurements of right and left ventricular volumes and function, degree of pulmonary valve regurgitation and tricuspid regurgitation. In addition, cardiopulmonary exercise test results, cardiac magnetic resonance imaging results, and in the latter period of the study knowledge-based reconstruction of right ventricular volumes using real-time three-dimensional echocardiographic analysis (VentriPoint) was added to the routine investigations. Peri-operative data included age at operation, size of the Trifecta St. Jude Medical® implanted and z-score compared with the native PV size, indexed effective orifice area of the implanted valve compared with the native PV, additional intra-operative procedures, site of cannulation for cardio-pulmonary bypass duration, aortic cross-clamp duration when used, and right ventricular remodelling. Immediate post-operative data included duration of mechanical ventilation, duration of intensive care unit and hospital stay, complications, and status on hospital discharge. Post-operative follow-up data included date of last follow-up, NYHA classification, results of echocardiography with measurements of the peak velocity through the implanted PV and presence and degree of regurgitation, cardiac magnetic resonance imaging and VentriPoint with their comparison with the pre-operative data relative to right ventricular volumes and function.

### 3.3. Statistical analysis

Hospital mortality was calculated from the date of the operation to 30 days post-operatively. Survival was calculated from the date of the operation until the last day of follow-up or date of death, whichever came first. Follow-up was calculated from the date of the operation to the date of last review clinic appointment. Z-scores were calculated for the size and the indexed effective orifice area of the Trifecta St. Jude Medical® implanted and compared with the native PV size.

Student’s *t*-tests were performed to evaluate the statistical difference, and a *p* < 0.05 was accepted to be statistically significant.

## 4. Results

### 4.1. Pre-operative data

Over the four-year period 71 patients underwent PV implantation of a Trifecta St. Jude Medical® for PV regurgitation. Their mean (± standard deviation) age was 24 ± 13 years (range four to 54 years), the mean (± SD) body weight was 62 ± 23 kg (range 15–112 kg). Their mean age at previous surgery was 3.2 ± 7.2 years (one month to 49 years), and surgery consisted of repair of tetralogy of Fallot in 83% (59/71), surgical pulmonary valvotomy or valvectomy in 11% (8/71), and relief of right ventricular outflow tract obstruction in 6% (4/71). The mean (± SD) interval between the first PV surgery and PV implantation was 21 ± 10 years (range two to 47 years). Pre-operative echocardiography confirmed the majority of patients (=97%, 69/71) to have severe degree of PV regurgitation, with the remaining 3% (2/71) suffering moderate regurgitation, with associated right ventricular outflow tract obstruction. The magnetic resonance imaging and knowledge-based reconstruction 3D volumetry (VentriPoint) showed a mean (± SD) PV regurgitation of 42 ± 9% (range 20–58%), mean indexed right ventricular end-diastolic volume of 169 ± 33 (range 130–265) ml m^–2^ BSA and mean ejection fraction of 46 ± 8% (range 33–61%). The cardio-pulmonary exercise tests showed a mean (± SD) peak O_2_/uptake of 24 ± 8 ml kg^–1^ min^–1^ (range 14–45 ml kg^–1^ min^–1^), predicted max O_2_/uptake 66 ± 17% (range 26–97%). The pre-operative NYHA class was I in 17% (12/71) patients, II in 70% (50/71) and III in 13% (9/71).

### 4.2. Intra-operative data

The mean duration of cardio-pulmonary bypass was 95 ± 30ʹ (range 38–190ʹ). Aortic cross-clamp was used in 23% (16/71) patients, with mean duration of 46 ± 31ʹ (range 8 to 95ʹ), with 77% (55/71) implantations with beating heart, without aortic cross-clamp. The size of implanted PV was 21 mm in seven patients, 23 mm in 33, 25 mm in 23, and 27 mm in eight. The *z*-score of the implanted PV was −0.16 ±0.80 (range −1.6 to 2.5). The effective orifice area indexed for B.S.A. of the implanted PV was 1.2 ± 0.3 (range 0.76 to −2.5) versus the effective orifice area indexed for B.S.A. of the native PV 1.5 ± 0.2 (range 1.2 to –2.1). Surgical right ventricular remodelling was associated in 76% (54/71) patients.

### 4.3. Post-operative data

There were no hospital deaths. The mean duration of mechanical ventilation was 6 ± 5 h (range 0–26 h), the mean stay in intensive care unit was 21 ± 11 h (range 12–64 h), the mean hospital stay was 6 ± 3 days (range three to 19 days). In a mean follow-up of 25 ± 14 months (range six to 53 months) there were no late deaths, no need for cardiac interventional procedures or re-operations, no valve-related complications, thrombosis or endocarditis. The last echocardiography showed absent PV regurgitation in 87.3% (62/71) patients, trivial/mild degree in 11.3% (8/71), and moderate degree in 1.4% (1/71). The mean max peak velocity through the right ventricular outflow tract was 1.6 ± 0.4 m s^–1^ (range 1.0–2.4 m s^–1^). The mean indexed right ventricular end-diastolic volume at knowledge-based reconstruction 3D volumetry (VentriPoint) was 96 ± 20 (range 63–151) ml m^–2^ BSA, significantly lower than the pre-operative value (*p* < 0.001), and the mean ejection fraction was 55 ± 4% (range 49–61%), significantly higher than the pre-operative value (*p* < 0.05). Almost all patients (99% = 70/71) remain in NYHA class I, with only 1% (1/71) in class II.

## 5. Discussion

Class I evidence indication for PV implantation based on the current European [20] and North-American [21] guidelines is the presence of symptoms. These guidelines are in agreement with the clinical indications generally accepted in the literature for symptomatic patients, with the support of the clinical non-invasive and invasive investigations.[6,8,10,14–19,22–25] The criteria for indications for PV implantation in asymptomatic patients, and particularly the choice of the best timing, are less clearly defined.[52] General agreement exists on the indication for PV implantation in asymptomatic patients in the presence of any of the following criteria, as judged by echocardiography and/or magnetic resonance imaging: (a) PV regurgitation >20%; (b) indexed end-diastolic right ventricular volume >120–150 ml m^–2^ BSA; or (c) indexed end-systolic right ventricular volume >80–90 ml m^–2^ BSA.[14,18,19,22,24,25] In our experience, all asymptomatic patients were matching all the accepted criteria of indication for PV implantation. The only two patients with indexed right ventricular end-diastolic volume < 150 ml m^–2^ BSA (respectively 130 and 142 ml m^–2^ BSA) were two of the four patients with associated right ventricular outflow tract obstruction.

The choice of the approach, between percutaneous interventional [6,26–33] versus surgical [8,10,12–19,34–38] PV implantation, was always the result of a multi-disciplinary meeting involving the interventional cardiologists as well as the congenital cardiac surgeons, with the decision making process taking into consideration: (a) the morphology and the size of the right ventricular outflow tract, in particular the aspect of the previously implanted transannular patch; (b) the morphology and the size of the pulmonary arteries if an enlargement or a reconstruction was required; (c) the presence of residual intracardiac defects requiring surgical repair; (d) the presence of extremely dilated right ventricle suggesting the association of surgical remodelling.

Regarding the choice of the most suitable valve, between the available mechanical and biological prostheses, our preference was not for the former, despite the proven longevity, because of the risks of thromboembolic phenomena and the requirement for life-long anticoagulation.[36,42] Our choice for a biological valve was in agreement with the vast majority of the reported studies, where tissue valves currently form the mainstay of the surgical treatment for patients with PV regurgitation.[3,34–41,43–48] Despite the numerous biological valves available for implantation in PV position, the optimal valve choice remains unknown, partly because long-term outcomes are deficient owing to structural valve deterioration necessitating further treatment.[43]

We decided to use the Trifecta St. Jude Medical® aortic valve instead of the other biological prostheses available because of several reasons: (a) the very good results obtained in aortic position, therefore with exposure to a much higher systolic and diastolic pressures, in long-term follow-up;[44–46] (b) the structure of the valve, with posts totally covered by pericardium, reducing the risk of embolism and haemolysis; (c) the availability in various sizes, compatible even for patients of large body weight.

The Trifecta St. Jude Medical® aortic valve is a trileaflet stented pericardial valve designed for implantation in aortic position, with a supra-annular sewing cuff with pericardial-covered posts as opposed to cloth.[46] Furthermore, its pericardial leaflets take their origins from the exterior of the valve construct, increasing the effective orifice area for any external diameter.[47]

In fact this favourable effective orifice area has been recently shown to provide a lower transvalvular pressure gradient across the Trifecta St. Jude Medical® aortic valve implanted in PV position in comparison with other biological prostheses,[44,45] and this further validated our choice of this type of valve, without need for any modification for implant in pulmonary valve position.

We expect that this characteristic may be associated with a reduced rate of structural valve deterioration over time, and this could be a very important potential advantage, since previous studies demonstrated that PV implantation performed at a young age with relatively smaller size biological valves correlated with a higher risk of valve failure and need for early valve re-replacement.[40,41]

Regarding the size of the valve to implant in PV position, our choice was to implant a relatively large size of the biological prosthesis, adequate for the effective orifice area of the native PV indexed for the patient BSA. This choice presents the advantage not only of avoiding a right ventricular outflow tract obstruction, but also of allowing for future percutaneous valve-in-valve implantation in the case of degeneration of the current valve. Of course we very carefully avoided oversizing the valve, because of the well-known reduced duration of oversized biological valves.

Our initial results corroborate the choice of Trifecta St. Jude Medical® aortic valve as a safe and effective biological valve for implantation in PV position.

In our experience surgical right ventricular modelling was associated in 76% patients at the time of PV implantation, to reduce the right ventricular volume and therefore contribute to improve the function.[49,50]

Contradictory results have been previously reported regarding the usefulness of the associated surgical right ventricular remodelling.[49–51] The negative results may have been due to the fact that the reported follow-up was too short to allow the full manifestation of the remodelling to be appreciated, and/or that the selected patients had irreversible right ventricular dilatation accounting for the lack of response to the surgical remodelling.[51] Our results with significant reduction of the right ventricular volumes immediately after surgery, particularly associating the right ventricular remodelling in the presence of sever right ventricular dilatation and/or dysfunction, are in favour of continuing to associate right ventricular remodelling in the presence of extremely dilated right ventricles, particularly with non-contractile portions.[49,50] In this regard, the current availability of the knowledge-based reconstruction of right ventricular volumes using real-time three-dimensional echocardiographic analysis (VentriPoint), and the proven very good correlation with the calculation of right ventricular volumes by magnetic resonance imaging,[53] is providing the possibility of serial evaluations of the right ventricular volumes and function. This will allow in the future an easier method to confirm the usefulness of associating surgical right ventricular remodelling.

### 5.1. Limits of the study

This study suffers from the following limitations: (a) it was a single-centre study; (b) it was a retrospective study, at least for the initial period; (c) the timings of the late post-operative echocardiogram and magnetic resonance imaging scans were not standardized at set time points; (d) follow-up was limited to the medium term.

## 6. Conclusions

The results of this study allow us the following conclusions: (a) the Trifecta St. Jude Medical® aortic valve is implantable in PV position with an easy and reproducible surgical technique; (b) a valve size adequate for patient BSA can be implanted with simultaneous RV remodelling; (c) the medium-term outcomes are good with maintained PV function, significantly reduced right ventricular volumes and significantly improved function; (d) the implantation of an adequate valve size will allow later percutaneous valve-in-valve implantation.
